# Complete Genome Sequences of Mycobacteriophages SynergyX, Abinghost, Bananafish, and Delton

**DOI:** 10.1128/mra.00286-22

**Published:** 2022-07-11

**Authors:** Laricca Y. London, Mary A. Ayuk, Amia C. Black, Laraine Cheung, Delandra M. Robinson, D’nai N. Thomas, Marcie H. Warner, Adrian D. Allen, Somiranjan Ghosh, Ayele Gugssa, Hemayet Ullah, Glory B. Bassey, Lourds M. Fernando, Madison M. Moore, Jerome J. Oliver, Esohe G. Irabor, Swagota D. Roy, Benedict K. Quagraine, Michael Smith, Howard University SEA-PHAGES Students, Winston A. Anderson, Courtney J. Robinson

**Affiliations:** a Department of Biological and Environmental Sciences, Alabama A&M University, Normal, Alabama, USA; b Department of Biology, Howard University, Washington, DC, USA; c Department of Biological Sciences, University of Pittsburgh, Pittsburgh, Pennsylvania, USA; Loyola University Chicago

## Abstract

Four lytic mycobacteriophages, namely, SynergyX, Abinghost, Bananafish, and Delton, were isolated from soil in Washington, DC, using the bacterial host Mycobacterium smegmatis mc^2^155. Analysis of the genomes revealed that they belong to two subclusters of actinobacteriophage cluster B (subclusters B2 and B3) and subcluster D1 of cluster D.

## ANNOUNCEMENT

Mycobacteriophages SynergyX, Abinghost, Bananafish, and Delton were isolated from soil samples collected from the campus of Howard University in Washington, DC (SynergyX, 38.9217N, 77.01917W; Abinghost, 38.9206N, 77.01694W; Bananafish, 38.9227N, 77.0208W; Delton, 38.9189N, 77.0183W). Students in the Science Education Alliance-Phage Hunters Advancing Genomics and Evolutionary Science (SEA-PHAGES) program followed the SEA-PHAGES Discovery Guide to isolate viruses with the goal of investigating bacteriophage evolution and diversity (1, 2). The samples were shaken at 37°C in 7H9 broth for 2 h, and a soil filtrate was generated as described previously (3). The filtrate was incubated with Mycobacterium smegmatis mc^2^155 cells at 37°C for 48 h under aerobic conditions. This enriched sample was filtered (0.22 μm) and plated with M. smegmatis mc^2^155 cells. Three rounds of purification were conducted for each resulting plaque of interest. Genomic DNA was extracted from high-titer lysates using the Promega Wizard DNA cleanup system (2). An Ultra II FS kit with dual-indexed barcoding (New England Biolabs) was used to generate genomic DNA libraries. Pooled libraries were sequenced using an Illumina MiSeq system to yield 150-base single-end reads (SynergyX, 232,719 reads; Abinghost, 356,927 reads; Bananafish, 382,199 reads; Delton, 264,896 reads). Reads were trimmed and assembled using Newbler v2.9 with default settings, yielding a single contig for each genome. Consed v29 was used to check contigs for completeness and accuracy and to determine phage termini (4–6). The phages had circularly permuted genomes.

The genomes were annotated using the Phage Evidence Collection and Annotation Network (PECAAN) (https://discover.kbrinsgd.org) and DNA Master 5.23.3 (http://cobamide2.bio.pitt.edu/computer.htm). Gene presence, start sites, and putative functions were determined using GLIMMER v3.0, GeneMark v2.5, Starterator v1.1 (https://github.com/SEA-PHAGES/starterator), Phamerator Actino_Draft v402 (https://phamerator.org), BLAST v2.11.0+, PhagesDB, HHpred 3.0, and the NCBI Conserved Domain Database (CDD) v3. ARAGON v1.2.38 and tRNAscan-SE v2.0 were used to detect tRNAs (7–14). PhagesDB and the NCBI SRA Taxonomy Analysis tool were used to assign taxonomy and cluster affiliations based on nucleotide similarity (13, 15). Default parameters were used for all software. Descriptive information for each genome is available in Table 1.

**TABLE 1:** Genome characteristics and accession numbers for four novel mycobacteriophages

Phage	Sequencing coverage (×)	GenBank accession no.	SRA accession no.	Subcluster	Genome size (bp)	G+C content (%)	No. of protein coding genes	Top BLASTn match (GenBank accession no.)	Identity to top BLASTn match (%)
SynergyX	478	MN444872	SRX10013849	B3	68,980	67.5	104	OrangeOswald (KR080203.1)	99.34
Abinghost	734	MN444873	SRX10013839	B3	68,634	67.6	103	Kamiyu (JN699018.1)	98.25
Bananafish	817	MK814751	SRX10013840	B2	67,366	68.9	93	Calamitous (MZ747518.1)	99.08
Delton	580	MK559427	SRX10013860	D1	64,963	59.6	87	Helpful (MN284908.1)	99.37

Nucleotide similarity suggested that the phages are members of the *Siphoviridae* family and that three of the phages should be assigned to cluster B, namely, SynergyX (subcluster B3), Abinghost (subcluster B3), and Bananafish (subcluster B2). Delton was affiliated with cluster D (subcluster D1) (16). The genome sizes (64,963 to 68,980 bp) and G+C content values (59.6% to 68.9%) were consistent with those of other members of the same subclusters, based on information available in PhagesDB (13). No tRNAs were detected. The phages were confirmed to be lytic based on the generation of clear plaques upon infection of the host, the presence of lysis system genes, and the absence of lysogeny genes. The lysis system includes lysin A and holin in Bananafish and lysin A and lysin B in SynergyX, Abinghost, and Delton. Also present are genes that encode structural proteins and proteins that play roles in DNA synthesis, modification, packaging, and repair processes.

### Data availability.

Complete genome sequences are available in GenBank, and raw sequencing reads are available in the NCBI SRA. Accession numbers are provided in Table 1.

## References

[1] Jordan TC, Burnett SH, Carson S, Caruso SM, Clase K, DeJong RJ, Dennehy JJ, Denver DR, Dunbar D, Elgin SCR, Findley AM, Gissendanner CR, Golebiewska UP, Guild N, Hartzog GA, Grillo WH, Hollowell GP, Hughes LE, Johnson A, King RA, Lewis LO, Li W, Rosenzweig F, Rubin MR, Saha MS, Sandoz J, Shaffer CD, Taylor B, Temple L, Vazquez E, Ware VC, Barker LP, Bradley KW, Jacobs-Sera D, Pope WH, Russell DA, Cresawn SG, Lopatto D, Bailey CP, Hatfull GF. 2014. A broadly implementable research course in phage discovery and genomics for first-year undergraduate students. mBio 5:e01051-13. doi:10.1128/mBio.01051-13. 24496795 PMC3950523

[2] Poxleitner M, Pope W, Jacobs-Sera D, Sivanathan V, Hatfull GF. 2018. Phage discovery guide. Howard Hughes Medical Institute, Chevy Chase, MD.

[3] London LY, Ayuk MA, Effiom D, Fashina F, Louis BJ, Tolsma SS, Allen AD, Dickson LA, Ghosh S, Gugssa A, Ullah H, Bassey GB, Fernando LM, Moore MM, Oliver JJ, Irabor EG, Roy SD, Quagraine BK, Smith M, Howard University SEA-PHAGES Students, Anderson WA, Robinson CJ. 2021. Complete genome sequences of mycobacteriophages Dallas and Jonghyun. Microbiol Resour Announc 10:e00304-21. doi:10.1128/MRA.00304-21. PMC8265233 34236221

[4] Margulies M, Egholm M, Altman WE, Attiya S, Bader JS, Bemben LA, Berka J, Braverman MS, Chen YJ, Chen Z, Dewell SB, Du L, Fierro JM, Gomes XV, Godwin BC, He W, Helgesen S, Ho CH, Irzyk GP, Jando SC, Alenquer MLI, Jarvie TP, Jirage KB, Kim JB, Knight JR, Lanza JR, Leamon JH, Lefkowitz SM, Lei M, Li J, Lohman KL, Lu H, Makhijani VB, McDade KE, McKenna MP, Myers EW, Nickerson E, Nobile JR, Plant R, Puc BP, Ronan MT, Roth GT, Sarkis GJ, Simons JF, Simpson JW, Srinivasan M, Tartaro KR, Tomasz A, Vogt KA, Volkmer GA, Wang SH, Wang Y, Weiner MP, Yu P, Begley RF, Rothberg JM. 2005. Genome sequencing in microfabricated high-density picolitre reactors. Nature 437:376–380. doi:10.1038/nature03959. 16056220 PMC1464427

[5] Gordon D, Green P. 2013. Consed: a graphical editor for next-generation sequencing. Bioinformatics 29:2936–2937. doi:10.1093/bioinformatics/btt515. 23995391 PMC3810858

[6] Russell DA. 2018. Sequencing, assembling, and finishing complete bacteriophage genomes. Methods Mol Biol 1681:109–125. doi:10.1007/978-1-4939-7343-9_9. 29134591

[7] Altschul SF, Gish W, Miller W, Myers EW, Lipman DJ. 1990. Basic local alignment search tool. J Mol Biol 215:403–410. doi:10.1016/S0022-2836(05)80360-2. 2231712

[8] Delcher AL, Bratke KA, Powers EC, Salzberg SL. 2007. Identifying bacterial genes and endosymbiont DNA with Glimmer. Bioinformatics 23:673–679. doi:10.1093/bioinformatics/btm009. 17237039 PMC2387122

[9] Besemer J, Borodovsky M. 2005. GeneMark: web software for gene finding in prokaryotes, eukaryotes and viruses. Nucleic Acids Res 33:W451–W454. doi:10.1093/nar/gki487. 15980510 PMC1160247

[10] Laslett D, Canback B. 2004. ARAGORN, a program to detect tRNA genes and tmRNA genes in nucleotide sequences. Nucleic Acids Res 32:11–16. doi:10.1093/nar/gkh152. 14704338 PMC373265

[11] Marchler-Bauer A, Derbyshire MK, Gonzales NR, Lu S, Chitsaz F, Geer LY, Geer RC, He J, Gwadz M, Hurwitz DI, Lanczycki CJ, Lu F, Marchler GH, Song JS, Thanki N, Wang Z, Yamashita RA, Zhang D, Zheng C, Bryant SH. 2015. CDD: NCBI’s Conserved Domain Database. Nucleic Acids Res 43:D222–D226. doi:10.1093/nar/gku1221. 25414356 PMC4383992

[12] Söding J, Biegert A, Lupas AN. 2005. The HHpred interactive server for protein homology detection and structure prediction. Nucleic Acids Res 33:W244–W248. doi:10.1093/nar/gki408. 15980461 PMC1160169

[13] Russell DA, Hatfull GF. 2017. PhagesDB: the Actinobacteriophage Database. Bioinformatics 33:784–786. doi:10.1093/bioinformatics/btw711. 28365761 PMC5860397

[14] Lowe TM, Eddy SR. 1997. tRNAscan-SE: a program for improved detection of transfer RNA genes in genomic sequence. Nucleic Acids Res 25:955–964. doi:10.1093/nar/25.5.955. 9023104 PMC146525

[15] Katz KS, Shutov O, Lapoint R, Kimelman M, Brister JR, O'Sullivan C. 2021. STAT: a fast, scalable, MinHash-based *k*-mer tool to assess Sequence Read Archive next generation sequence submissions. Genome Biol 22:270. doi:10.1186/s13059-021-02490-0. 34544477 PMC8450716

[16] Mavrich TN, Hatfull GF. 2017. Bacteriophage evolution differs by host, lifestyle and genome. Nat Microbiol 2:17112. doi:10.1038/nmicrobiol.2017.112. 28692019 PMC5540316

